# The Experience of Health Care Workers Caring for Older Adults During the COVID-19 Pandemic

**DOI:** 10.1093/geroni/igab046.1472

**Published:** 2021-12-17

**Authors:** Tiffany Washington, Terri Lewinson, Jennifer Craft Morgan

## Abstract

Older adults are at increased risk for COVID-19 illness, hospitalization, and mortality. Essential health care workers became the backbone of their care during the pandemic, and their experiences are worthy of discussion. This symposium will highlight the emotional impact of COVID-19 on health care workers, and their scope of practice in various health settings. Using data from a cross-sectional survey, presenter one will describe concerns and coping strategies among nursing home social workers during COVID-19. Next, presenter two will present findings on the emotional health and wellbeing of home care workers. Then, presenter three will describe the experience of the VA’s strike teams sent into 82 Florida nursing homes most impacted by COVID. Presenter four will describe findings from a qualitative study on emotional distress experienced by hospital-based medical directors, nurse practitioners, and other health care workers. Finally, presenter five will describe findings from a scope of practice among health care social workers study, with an emphasis on how policies in their respective settings impacted their work. Taken together, the presenters’ findings in this symposium have implications for practice and policy recommendations to improve the experience of health care workers during pandemics.

